# Palmitic and oleic acids induce macrophage foam cell formation through C/EBPβ activation

**DOI:** 10.3389/fimmu.2026.1809059

**Published:** 2026-04-29

**Authors:** Gang Wang, Yifan Zhu, Yang Liu, Wei Huang, Yihua Wei, Yuan Liu, Binbin Liu, Li Su

**Affiliations:** 1 Department of Cardiology, Affiliated Banan Hospital of Chongqing Medical University, Chongqing, China; 2 Department of Cardiology, The Second Affiliated Hospital of Chongqing Medical University, Chongqing, China; 3 Xiamen Cardiovascular Hospital, School of Medicine, Xiamen University, Xiamen, Fujian, China; 4 Department of Traditional Chinese Medicine, School of Medicine, Xiamen University, Xiamen, Fujian, China; 5 Department of Traditional Chinese Medicine, The First Affiliated Hospital of Guangxi Medical University, Nanning, Guangxi, China

**Keywords:** C/EBPβ, CD36, dietary fatty acids, epigenetic regulation, foam cell formation

## Abstract

**Background:**

Foam cell formation is a critical early event in atherosclerosis. While oxidized low-density lipoprotein (oxLDL)-driven mechanisms are well studied, the contribution of free fatty acids (FFAs), particularly under high-fat dietary intake, is less defined. CCAAT/enhancer-binding protein β (C/EBPβ), a transcription factor regulating macrophage lipid metabolism, has been implicated, but its epigenetic role in FFA-induced lipid accumulation remains unclear.

**Methods:**

We exposed RAW 264.7 macrophages and mouse bone marrow-derived macrophages to palmitic and oleic acids to model FFA-driven foam cell formation *in vitro*. Multi-omics approaches, including RNA sequencing and assay for transposase-accessible chromatin using sequencing (ATAC-seq), were applied to assess transcriptional and chromatin changes, and C/EBPβ deficiency was used to test its functional relevance. Lipid accumulation and CD36 expression were evaluated by BODIPY staining, flow cytometry, and quantitative real-time polymerase chain reaction (qRT-PCR).

**Results:**

Exposure to palmitic and oleic acids markedly increased intracellular lipid content and induced upregulation of C/EBPβ and CD36. Chromatin profiling revealed C/EBPβ-dependent accessibility at promoters of genes involved in f atty acids uptake and storage. Silencing of C/EBPβ significantly reduced CD36 expression and lipid accumulation, attenuating foam cell formation.

**Conclusion:**

These findings establish a novel C/EBPβ–CD36 regulatory axis that drives FFA-induced foam cell formation through epigenetic remodeling. This mechanism provides new insight into dietary fatty acids–mediated macrophage reprogramming and suggests potential therapeutic targets for early atherosclerosis and lipid-driven cardiovascular disease.

## 1 Introduction

Foam cells are characterized by the accumulation of lipid-rich droplets in their cytoplasm, giving them a distinctive foam-like appearance. These cells localize beneath the endothelial layer of blood vessels, where they secrete pro-inflammatory mediators and degrade the extracellular matrix of atherosclerotic plaques, contributing to plaque formation, destabilization, and eventual rupture (1, 2). These pathological processes ultimately increase the risk of cardiovascular events. Currently, there is a lack of insight into the pathways involved in the uptake of certain types of lipids by macrophages, thus limiting our understanding of the mechanisms leading to the appearance of foamy cells (3, 4). While foam cell formation driven by oxidized low-density lipoprotein (oxLDL) has been extensively studied in advanced atherosclerotic lesions (5, 6), emerging evidence suggests that metabolic lipid overload caused by elevated circulating free fatty acids (FFAs) may drive a distinct pathogenic mechanism, particularly during the early stages of atherosclerosis (7–10).

Fatty acids serve as vital energy substrates for macrophages, fueling ATP production to support essential cellular functions and maintain immunometabolic homeostasis (11, 12). Under physiological conditions, lipid uptake by macrophages is a normal and adaptive process observed in various tissues, such as adipose tissue, liver, and lungs. However, under pathological conditions—including atherosclerosis, non-alcoholic fatty liver disease (NAFLD), and obesity—this adaptive mechanism becomes dysregulated. Excessive lipid influx overwhelms the macrophages’ metabolic capacity, resulting in pathological lipid accumulation and foam cell formation that actively contribute to disease progression (13–15). Importantly, different lipid subtypes influence macrophage lipid handling—including uptake, storage, and efflux—via distinct molecular pathways (4, 16).

In recent decades, the widespread adoption of Western-style diets—characterized by excessive intake of free fatty acids, refined sugars, and ultra-processed foods—has been closely associated with a global rise in obesity, insulin resistance, NAFLD, and cardiovascular disorders (17–19). High dietary loads of FFAs, especially palmitic acid, induce metabolic stress in various tissues, including the vascular wall (10). This nutritional overload directly impacts macrophage immunometabolism, promoting lipid accumulation and inflammatory activation (9, 19, 20). Notably, increasing evidence suggests that FFA-induced lipid loading may promote macrophage foam cell formation independently of oxLDL, highlighting the need to better understand the molecular mechanisms underlying FFA-driven foam cell formation.

Epigenetic regulation has emerged as a critical determinant of macrophage phenotypic plasticity in the context of foam cell formation (21). In oxLDL-induced foam cells, elevated trimethylation of histone 3 lysine 4—a transcriptionally active histone mark—at pro-inflammatory gene promoters enhances cytokine expression and promotes foam cell development (22). Regulatory factor X, a transcription factor, has been shown to inhibit foam cell formation and attenuate plaque progression in murine models of atherosclerosis by directly repressing CD36 transcription in macrophages (23). However, the epigenetic mechanisms specifically governing FFA-driven foam cell formation remain poorly understood.

CCAAT/enhancer-binding protein β (C/EBPβ), a member of the C/EBP family of transcription factors, has emerged as a pivotal regulator of macrophage lipid metabolism (8, 24). Macrophages deficient in C/EBPβ exhibit impaired clearance of surfactant lipids in the pulmonary alveoli, resulting in a pulmonary alveolar proteinosis-like phenotype (15). Moreover, C/EBPβ-deficient macrophages show significantly reduced inflammation, cholesterol accumulation (25–29). These findings underscore the essential role of C/EBPβ in facilitating lipid uptake and promoting foam cell formation in macrophages. However, the epigenetic mechanisms through which C/EBPβ regulates FFA-induced lipid accumulation and foam cell formation remain largely undefined. Meanwhile, the scavenger receptor CD36 is known to play a critical role in mediating fatty acids uptake in macrophages (30, 31). Interestingly, unsaturated fatty acids have been shown to induce CD36 gene expression in human macrophages in a dose-dependent manner, suggesting that fatty acids upregulate the mechanisms required for their cellular uptake (32). Nevertheless, the molecular mechanisms by which macrophages utilize CD36 to take up FFAs—particularly in the context of foam cell formation—are still poorly understood.

In this study, we demonstrate that palmitic and oleic acids stimulated RAW 264.7 macrophages and mouse bone marrow-derived macrophages undergo C/EBPβ-dependent chromatin remodeling at loci associated with fatty acids metabolism, orchestrating the epigenetic activation of lipid metabolic pathways. Through integrative multi-omics analysis, C/EBPβ is identified as a central regulatory hub linking chromatin remodeling to transcriptional reprogramming. Deficiency of C/EBPβ markedly suppresses lipid accumulation, establishing its essential role in mediating palmitic and oleic acids induced foam cell formation.

Our findings uncover a previously unrecognized epigenetic axis, C/EBPβ–CD36, that drives palmitic and oleic acids induced foam cell formation, and highlight potential therapeutic targets for the treatment of lipid metabolism–associated cardiovascular disease.

## 2 Methods

### 2.1 Preparation of reagents

To prepare 10 mM stock solutions of palmitic acid (PA) or oleic acid (OA) conjugated with bovine serum albumin (BSA), PA or OA powder was first dissolved in 1 mL of 0.1 M NaOH. The mixture was heated at 90 °C for 5 minutes, briefly vortexed, and then heated for an additional 5 minutes until the solution became clear.

Meanwhile, 0.5 g of BSA was dissolved in 9 mL of double-distilled water (ddH_2_O) and incubated in a 55 °C water bath until fully dissolved. The heated fatty acid solution was then rapidly added to the prewarmed BSA solution while maintaining the temperature at 55 °C, with immediate mixing to prevent precipitation.

The final working solution contained10 mM fatty acid in 5% BSA. The mixture was vortexed thoroughly, sterile-filtered using a 0.22 μm membrane filter, aliquoted, and stored at –20 °C until use. All reagents used in this study are listed in **Supplementary Table S1**.

### 2.2 Cell culture

RAW 264.7 macrophages (CL-0190, Procell, Wuhan, China), were cultured in Dulbecco’s Modified Eagle Medium (DMEM) supplemented with 10% fetal bovine serum (FBS), 100 U/mL penicillin, and 0.1 mg/mL streptomycin at 37 °C in a humidified atmosphere containing 5% CO_2_.

For gene knockout, RAW 264.7 cells were transduced with lentivirus carrying lentiCRISPR v2 plasmid encoding a guide RNA (gRNA) targeting *Cebpb* or an empty vector control using the Gene Pulser Xcell Eukaryotic System (Bio-Rad, 165-2661, CA, USA). Transfected cells were selected with puromycin (concentration and duration as optimized) for 3 days. The gRNA sequence targeting *Cebpb* is provided in **Supplementary Table S1**.

For functional assays, cells were treated with PA+OA, or the CD36 inhibitor sulfosuccinimidyl oleate (SSO) at the indicated concentrations and time points.

### 2.3 Mice studies

C57BL/6J male mice (8 weeks old, weighing 22–25 g) were obtained from GemPharmatech (Guangzhou, China). Myeloid-specific C/EBPβ knockout mice were generated by crossing *Cebpb^flox/flox^* mice with *Lyz2^Cre^* transgenic mice, yielding *Cebpb^flox/flox^ Lyz2^Cre^* mice. Littermate *Cebpb^flox/flox^* mice lacking Cre recombinase were used as controls. All mice were housed under specific pathogen-free (SPF) conditions at Xiamen University, maintained on a 12 h light/12 h dark cycle at 22 - 23 °C with free access to food and water. Bone marrow–derived macrophages were isolated from *Cebpb^flox/flox^* and *Cebpb^flox/flox^ Lyz2^Cre^* mice.

All animal experiments were conducted in accordance with the National Institutes of Health (NIH) Guidelines for the Care and Use of Laboratory Animals and were approved by the Animal Care and Protection Committee of Xiamen University (Protocol No. XMULAC20200150). This study is reported in accordance with the ARRIVE guidelines.

For tissue collection, eight-week-old male mice were anesthetized using isoflurane inhalation anesthesia until a surgical plane of anesthesia was achieved. Mice were then humanely euthanized by cervical dislocation under deep anesthesia, followed by bone marrow isolation, in accordance with institutional and ethical guidelines.

### 2.4 Isolation and differentiation of bone marrow–derived macrophages

Mice were sacrificed and briefly immersed in 75% ethanol for disinfection. The femurs and tibias were carefully dissected and cleaned of muscle and connective tissue under sterile conditions. Bones were initially rinsed in phosphate-buffered saline (PBS) containing 500 U/mL penicillin and 0.5 mg/mL streptomycin, on ice, then further cleaned in fresh PBS supplemented with 300 U/mL penicillin and 0.3 mg/mL streptomycin, to remove residual tissue. After a final rinse in PBS, both ends of the femur and tibia were cut, and the bone marrow was flushed out using a 1 mL syringe filled with RPMI-1640 medium supplemented with 10% FBS and 100 U/mL penicillin, 0.1 mg/mL streptomycin.

The flushed marrow cells were collected in a sterile dish and transferred into a 15 mL centrifuge tube. Cells were pelleted by centrifugation at 1000 rpm for 5 minutes, and the supernatant was discarded. The pellet was resuspended by red blood cell lysis using 3 mL of ACK lysis buffer on ice for 3 minutes. After centrifugation and resuspension, cells were passed through a 70 μm cell strainer and collected into a fresh 15 mL tube.

Cells were then resuspended in RPMI-1640 supplemented with 10% FBS, 100 U/mL penicillin, 0.1 mg/mL streptomycin, and 50 ng/mL macrophage colony-stimulating factor (M-CSF), and plated in 10 cm culture dishes. The medium was refreshed every 3 days. After 6 days of differentiation, adherent macrophages were gently detached using 10 mM EDTA in RPMI-1640 containing 1% FBS, and seeded into 12-well plates for downstream experiments. BMDMs were isolated from n = 3 individual male C57BL/6 mice, and all experiments were performed with 3 biological replicates, each consisting of an independently derived BMDM preparation.

### 2.5 Transcriptomics study

Total RNA extraction from RAW 264.7 macrophages was performed using Trizol-based isolation according to the supplier’s protocol. Complementary DNA synthesis and library construction were carried out with the NEBNext Ultra RNA Library Prep Kit designed for Illumina platforms. Pooled libraries underwent high-throughput sequencing on the Novaseq 6000 system (Illumina, CA, USA), yielding 150-bp paired-end sequencing data. RNA sequencing (RNA-seq) analysis was performed using 3 independent biological replicates per experiment condition and total of 12 experimental conditions were included in the analysis.

For genomic alignment, sequencing reads were processed using HISAT 2.2.1 software against the Mus musculus reference genome assembly GRCm39. Gene-level quantification was achieved through FeatureCounts, generating read count matrices for downstream analysis. Differential expression analysis was implemented in the R statistical environment, utilizing the Limma package to detect statistically significant gene expression changes.

### 2.6 ATAC-seq and chromatin immunoprecipitation sequencing study

For chromatin accessibility profiling, 5 × 10^4^ cells per sample were collected from independently prepared cultures (two independent biological replicates were performed per experiment condition). Cells underwent two washes in ice-cold phosphate-buffered saline (PBS) followed by resuspension in 50 μl of chilled lysis buffer containing 10 mM Tris-HCl (pH 7.4), 10 mM NaCl, 3 mM MgCl2, and detergents (0.1% Tween, 0.1% NP40, 0.01% Digitonin). Following centrifugation (750 × g, 10 min, 4 °C), isolated nuclei were subjected to transposition reaction in a 50 μl mixture containing 5 μl of Tn5 Transposase (TruePrep DNA Library Prep kit V2, Illumina) with incubation at 37 °C for 30 minutes. The resulting DNA fragments were purified using QIAGEN’s MiniElute purification system, followed by index labeling with Novo NGS Index Kit and amplification to 240 fmol concentration using NEBNext Ultra II Q5 Master Mix. Final PCR products were cleaned using a PCR purification system prior to paired-end sequencing on the Novaseq 6000 platform. ATAC-seq experiments were performed with two independent biological replicates per experiment condition, and a total of 4 conditions were analyzed.

Raw sequencing reads from ChIP-seq targeting C/EBPβ in macrophages were obtained from the GEO database (accession number: GSE173970) (15) and ATAC-seq. Quality trimming was performed using Trim Galore, and the cleaned reads were aligned to the mouse reference genome (mm10 assembly) with Bowtie2. PCR duplicates and multi-mapped reads were removed using Sambamba. Chromatin accessibility peaks were identified with the MACS2 peak-calling algorithm, and transcription factor binding motifs were characterized using the HOMER motif discovery pipeline.

Quantitative analysis was performed using DESeq2 package with normalization to reads per kilobase per million mapped reads (RPKM). Data visualization was achieved through Integrative Genomics Viewer (IGV), and transcription start site (TSS)-associated signal density profiles were generated using DeepTools computational suite.

### 2.7 Bioinformatic analysis of single-cell RNA sequencing

Single-cell RNA sequencing data of foam cells were obtained from the GEO database (accession number: GSE123587) (33) and analyzed using the Seurat R package. Initial quality control was performed by removing genes expressed in fewer than three cells, as well as excluding cells that met any of the following criteria: expression of fewer than 200 or more than 2,500 genes, or mitochondrial gene expression exceeding 5%.

### 2.8 Flow cytometry

For intracellular lipid staining, cultured cells were fixed in IC Fixation Buffer and stained with BODIPY (1:200 dilution) and anti-CD36 antibody (1:400 dilution) for 30 minutes at room temperature.

For peripheral blood analysis, freshly collected mouse blood was lysed in ACK lysis buffer on ice for 10 minutes, followed by centrifugation at 3000 rpm for 5 minutes to pellet the cells. After red blood cell lysis, cells were incubated with Fc receptor blocking antibody to minimize nonspecific binding. Surface staining was performed on ice for 30 minutes using fluorophore-conjugated antibodies in MACS buffer (1× PBS containing 1% BSA and 2 mM EDTA).

Samples were acquired on a FACS LSR Fortessa flow cytometer (Becton Dickinson, Franklin Lakes, NJ, USA), and data were analyzed using FlowJo software (Tree Star, USA). Doublets and multiplets were excluded by forward/side scatter gating, and non-viable cells were excluded using a fixable viability dye. Representative gating strategies are shown in **Supplementary Figure S1** and complete list of antibodies used is provided in **Supplementary Table S1**.

### 2.9 ChIP

Chromatin immunoprecipitation assays were carried out in RAW 264.7 cells following a modified protocol based on previous reports (34). Approximately 5 × 10^6^ cells were fixed with 1% formaldehyde at room temperature for 8 minutes to crosslink protein-DNA interactions, followed by quenching with 1.25 mM glycine for 5 minutes. Cells were lysed in a hypotonic swelling buffer (50 mM HEPES-KOH, pH 7.5, 140 mM NaCl, 1 mM EDTA, 10% glycerol, 0.5% NP-40, 0.25% Triton X-100, and protease inhibitors) for 10 minutes on ice to remove cytoplasmic content.

Nuclei were then isolated and incubated with lysis buffer (50 mM Tris-HCl, pH 8.0, 10 mM EDTA, 1% SDS, and protease inhibitors) for 10 minutes on ice. Chromatin was sheared by sonication (Bioruptor, Diagenode, Belgium) to achieve an average fragment size of ~500 bp. The sheared lysates were diluted tenfold in ChIP dilution buffer (10 mM Tris-HCl, pH 8.0, 100 mM NaCl, 1 mM EDTA, 0.5 mM EGTA, 1% Triton X-100, 0.1% sodium deoxycholate, and protease inhibitors), and immunoprecipitation was performed overnight using 3 µg of either anti-C/EBPβ antibody or control mouse IgG.

Protein-DNA complexes were captured with Protein G magnetic Dynabeads, followed by three washes with high-salt buffer (50 mM HEPES-KOH, pH 7.0, 500 mM LiCl, 1 mM EDTA, 0.7% sodium deoxycholate, 1% NP-40). Bound chromatin was eluted, crosslinks were reversed, and DNA was purified using the QIAquick PCR Purification Kit (Qiagen). Enrichment of target DNA sequences was analyzed by quantitative PCR using SYBR Green Master Mix and the following thermal cycling conditions: initial denaturation at 95 °C for 1 minute, followed by 40 cycles of 95 °C for 15 seconds, 60 °C for 20 seconds, and 72 °C for 40 seconds. Primer sequences used in this study are listed in **Supplementary Table S1**.

### 2.10 Quantitative real-time polymerase chain reaction

qRT-PCR was conducted using the RNA-direct SYBR Green Real-Time PCR Master Mix. Glyceraldehyde 3-phosphate dehydrogenase (GAPDH) was utilized as the internal control. The primer sequences are provided in **Supplementary Table S1**.

### 2.11 Immunofluorescence staining

To visualize intracellular lipid accumulation, cells were cultured on glass coverslips and fixed with IC fixation for 20 minutes at room temperature. After fixation, cells were rinsed twice with phosphate-buffered saline (PBS) and incubated with BODIPY (1:200 dilution) for lipid staining. Following three washes in PBS, coverslips were mounted, and fluorescence images were acquired using the EVOS FL Auto 2 imaging system.

For C/EBPβ and BODIPY double immunofluorescence staining, cells were seeded and treated on coverslips as described above. Cells were fixed with 4% Paraformaldehyde for 30 minutes, then permeabilized with 0.2% Triton X-100 in PBS for 10 minutes at room temperature. After three washes (5 minutes each) in PBS, cells were blocked in 5% bovine serum albumin (BSA) in PBS for 30 minutes to prevent nonspecific binding.

Primary antibody incubation was performed using anti-C/EBPβ antibody (1:300 dilution in 5% BSA in PBS) for 1 hour at room temperature. After washing, cells were incubated with Alexa Fluor 488-conjugated secondary antibody (1:800 dilution) and BODIPY simultaneously for 1 hour at room temperature. Nuclei were counterstained with DAPI, and coverslips were mounted using DAPI-containing mounting medium. Confocal images were captured using a Leica SP8 confocal microscope.

### 2.12 Western blot

Equal amounts of total protein were separated by SDS-PAGE and then electrophoretically transferred onto polyvinylidene difluoride (PVDF) membranes (Bio-Rad, Cat. #1620177) according to standard protocols. Membranes were blocked at room temperature for 2–3 hours using Tris-buffered saline containing 0.5% Tween-20 (TBST) and 3% non-fat milk to prevent nonspecific binding. Following blocking, membranes were incubated overnight at 4 °C with the appropriate primary antibodies diluted in TBST. The next day, after several washes in TBST, membranes were incubated with horseradish peroxidase (HRP)-conjugated secondary antibodies for 2–3 hours at room temperature. Immunoreactive protein bands were detected using the Western Bright enhanced chemiluminescence (ECL) HRP substrate (Advansta) and imaged with a Bio-Rad ChemiDoc MP imaging system. A complete list of primary and secondary antibodies used is provided in **Supplementary Table S1**. For presentation purposes, representative blots were cropped; full-length, uncropped blots are provided in **Supplementary Figure S2A**.

### 2.13 Statistical analysis

Statistical analysis was performed with Prism 9 software. Data are shown as mean ± standard error of the mean (SEM). Statistical tests performed for each dataset are described within the relevant figure legends. P-values ≤0.05 were considered statistically significant. P-values are denoted in figures as follows: ns, not significant, ^*^p < 0.05, ^**^p < 0.01, ^***^p < 0.001.

## 3 Result

### 3.1 Palmitic and oleic acids induce macrophage foam cell formation

**Figure 1:**
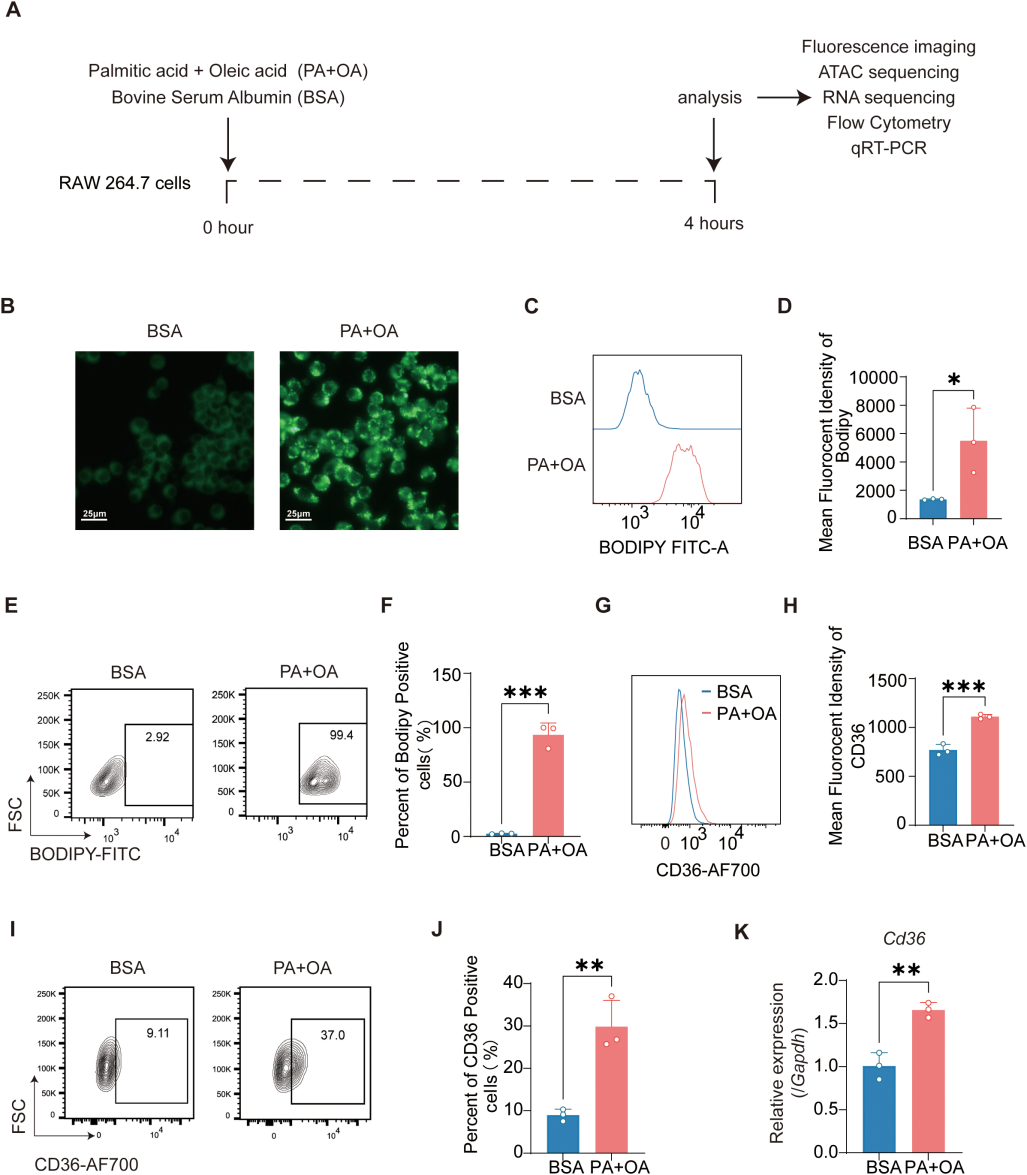
Palmitic and oleic acids induce foam cell formation in macrophages. **(A)** Schematic overview of the experimental design showing RAW 264.7 cell treatment with PA+OA or BSA, followed by indicated analyses. **(B)** Representative fluorescence images of BODIPY-stained RAW 264.7 cells after 4 h treatment with PA+OA or BSA. Scale bar, 25 μm. **(C, D)** Mean fluorescence intensity (MFI) of BODIPY in RAW 264.7 cells measured by flow cytometry (n = 3). **(E, F)** Percentage of BODIPY-positive RAW 264.7 cells determined by flow cytometry (n = 3). **(G, H)** MFI of CD36 in RAW 264.7 cells measured by flow cytometry (n = 3). **(I, J)** Percentage of CD36-positive RAW 264.7 cells determined by flow cytometry (n = 3). **(K)** Relative mRNA expression of *Cd36* in RAW 264.7 cells determined by qRT-PCR (n = 3). Data are presented as mean ± SEM. Statistical analysis was performed using an unpaired t-test. ^*^p < 0.05, ^**^p < 0.01, ^***^p < 0.001. Three independent experiments were performed.

To evaluate the effects of free fatty acids (FFAs) on foam cell formation, RAW 264.7 macrophages were treated with two of the most abundant dietary fatty acids—150 μM PA and 300 μM OA for 4 hours (**Figure 1A**). Lipid accumulation was visualized by BODIPY staining under fluorescence microscopy, revealing a marked increase in intracellular lipid droplets following PA+OA treatment (**Figure 1B**). To quantify this effect, flow cytometry analysis was performed. PA+OA exposure significantly increased the mean fluorescence intensity (MFI) of BODIPY (**Figures 1C, D**), with over 90% of cells exhibiting positive BODIPY signals (**Figures 1E, F**), indicating robust lipid accumulation in the majority of macrophages.

To assess the expression of CD36, a key transporter involved in fatty acid uptake, we examined both protein and mRNA levels. Western blot and flow cytometry analysis revealed a significant increase in CD36 protein expression after PA+OA stimulation (**Figures 1G–J**), as evidenced by elevated MFI and a higher percentage of CD36-positive cells. In parallel, *Cd36* mRNA levels were also significantly upregulated following fatty acid treatment (**Figure 1K**).

Taken together, these data demonstrate that the typical dietary fatty acids PA and OA markedly promote foam cell formation in macrophages, potentially via enhanced lipid uptake and CD36 upregulation.

### 3.2 Palmitic and oleic acid induced CD36-dependent foam cell formation

**Figure 2:**
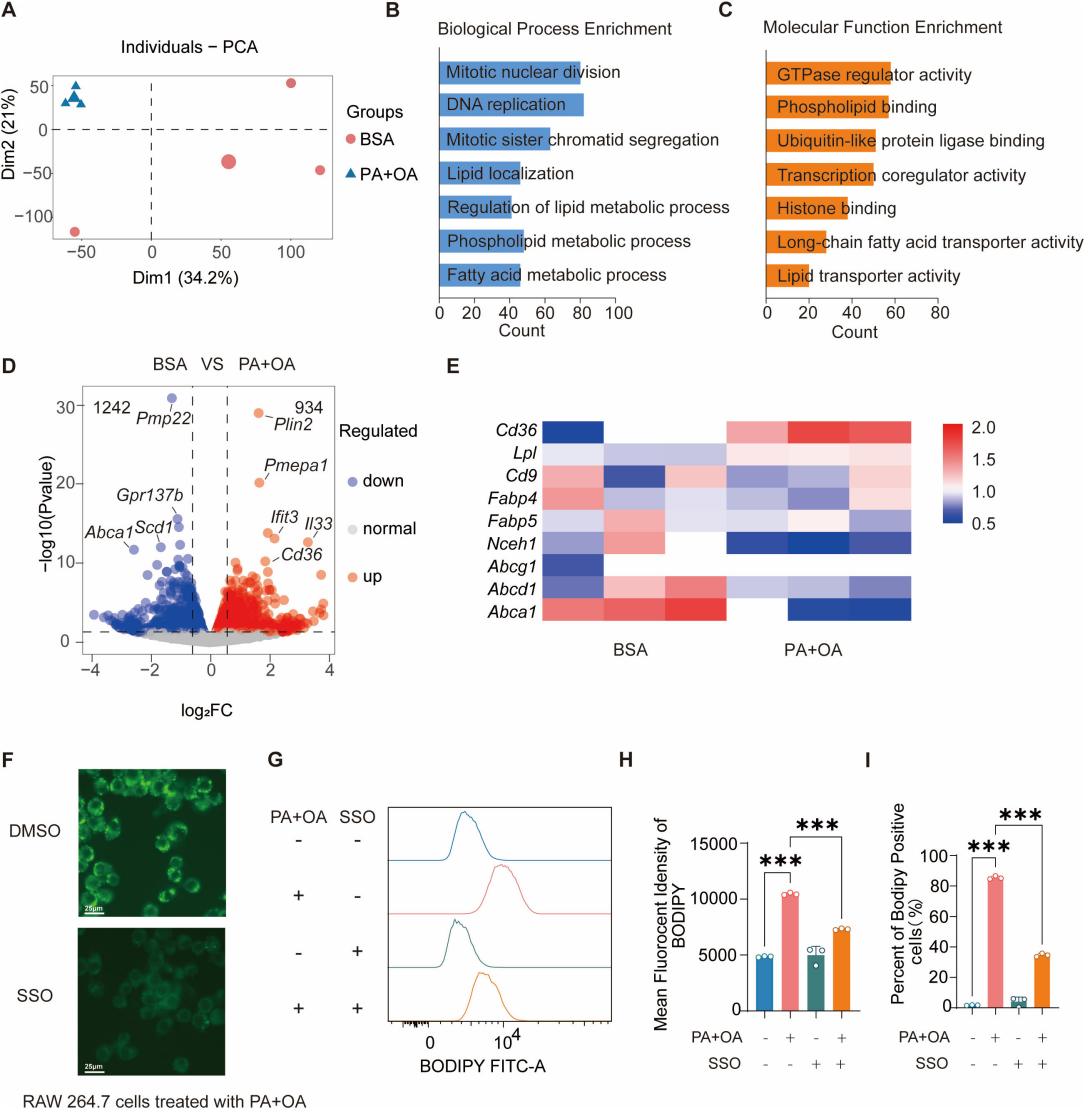
Palmitic and oleic acids induce CD36-dependent foam cell formation. **(A)** Principal component analysis (PCA) of RNA-seq data from RAW 264.7 cells treated with BSA or PA+OA. **(B, C)** Gene Ontology (GO) enrichment analysis of differentially expressed genes, showing enriched terms in the Biological Process **(B)** and Molecular Function **(C)** categories. **(D)** Volcano plot of differentially expressed genes between BSA- and PA+OA-treated RAW 264.7 cells. **(E)** Heatmap of fatty acid foam cell–related genes from RNA-seq analysis. **(F)** Representative fluorescence images of BODIPY-stained RAW 264.7 cells after 4 h treatment with PA+OA, in the presence of DMSO or the CD36 inhibitor SSO. Scale bar, 25 μm. **(G, H)** MFI of BODIPY in RAW 264.7 cells measured by flow cytometry (n = 3). **(I)** Percentage of BODIPY-positive RAW 264.7 cells determined by flow cytometry (n = 3). Data are presented as mean ± SEM. Statistical analysis was performed using one-way ANOVA with Tukey’s multiple comparisons test. ^***^p < 0.001. Three independent experiments were performed.

To further investigate the changes underlying PA+OA-induced foam cell formation, we performed RNA sequencing on RAW 264.7 macrophages treated with PA+OA or BSA control. Principal component analysis (PCA) and hierarchical clustering (**Figure 2A**) revealed clear transcriptomic separation between the treatment and control groups. Pathway enrichment analysis identified significant alterations in fatty acid metabolism and lipid transport pathways (**Figures 2B, C**).

The volcano plot showed 934 genes upregulated and 1242 genes downregulated upon PA+OA treatment, with a prominent increase in *Cd36* expression (**Figure 2D**). In addition to *Cd36*, several representative upregulated genes related to lipid metabolism and macrophage activation were identified, including *Plin2*, *Pmepa1*, *Ifit2*, and *Il33*. Notably, *Plin2* is associated with lipid droplet formation, indicating enhanced intracellular lipid storage (35), while *Ifit2* has been linked to lipid accumulation and impaired cholesterol efflux in atherosclerotic macrophages (36). Conversely, several genes involved in lipid homeostasis were downregulated, including *Abca1* and *Scd1*. As *Abca1* is a key regulator of cholesterol efflux, its reduction suggests impaired lipid export capacity (37), while decreased *Scd1* further indicates disrupted lipid metabolic balance (38). Collectively, these transcriptional changes—characterized by enhanced lipid storage and inflammatory activation alongside reduced cholesterol efflux, which support a shift toward lipid accumulation and foam cell formation.

A heatmap of selected genes related to lipid uptake and efflux (**Figure 2E**) revealed that, unlike *Cd36*, the expression levels of other fatty acid transporters such as *Fabp4* and *Fabp5* remained unchanged. Conversely, the cholesterol efflux transporter *Abca1* was notably downregulated. These findings suggest a shift in lipid handling, favoring enhanced uptake through CD36 and diminished efflux, thereby contributing to intracellular lipid accumulation and foam cell formation.

To validate the role of CD36 in this process, we employed the specific CD36 inhibitor sulfosuccinimidyl oleate (SSO). Treatment with 20 μM SSO markedly reduced PA+OA-induced lipid accumulation (**Figure 2F**), as evidenced by decreased BODIPY fluorescence intensity and a lower percentage of BODIPY-positive cells (**Figures 2G, H**).

Collectively, these results indicate that exposure to FFAs induces a transcriptomic reprogramming in macrophages that promotes lipid accumulation primarily via CD36-mediated uptake, underscoring the pivotal role of CD36 in foam cell formation.

### 3.3 Palmitic and oleic acids modulate epigenetic landscape alterations involving C/EBPβ

**Figure 3:**
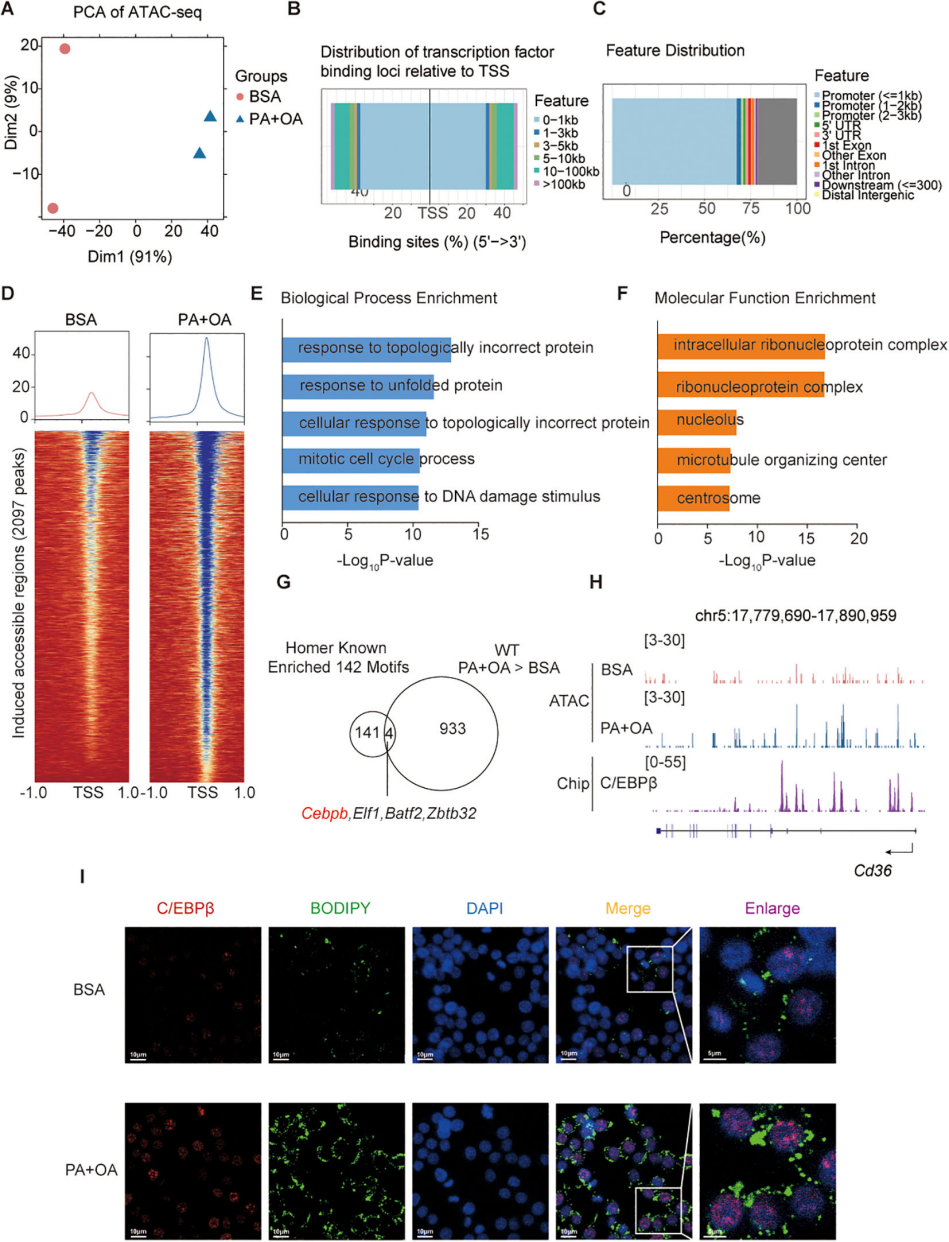
Palmitic and oleic acids modulate C/EBPβ-mediated epigenetic landscape alterations. **(A)** ATAC-seq was performed on RAW 264.7 cells treated with BSA or PA+OA for 4 h (n = 2). PCA plot of ATAC-seq data. **(B, C)** Peak distribution among genomic regions in individual samples. **(D)** Peak distribution of PA+OA-induced significantly altered chromatin accessibility regions. **(E, F)** GO enrichment analysis of PA+OA-induced significantly altered chromatin accessibility regions, showing enriched terms in the Biological Process **(E)** and Molecular Function **(F)** categories. **(G)** Venn diagram showing the overlap between genes identified by HOMER motif enrichment analysis of PA+OA-induced significantly altered chromatin accessibility regions and PA+OA-induced differentially expressed genes. **(H)** Representative ATAC-seq and ChIP-seq tracks for C/EBPβ on the *Cd36* locus. ChIP-seq data for C/EBPβ were obtained from GSE173970. **(I)** Representative fluorescence images of RAW 264.7 cells treated with BSA or PA+OA, stained for C/EBPβ (red), BODIPY (green), and counterstained with DAPI (blue).

To explore the epigenetic mechanisms underlying FFA-induced foam cell formation, we performed ATAC-seq on RAW 264.7 macrophages treated with PA+OA. PCA analysis revealed distinct shifts in the chromatin accessibility landscape following PA+OA exposure (**Figure 3A**). Notably, the open chromatin regions were predominantly enriched at promoter regions and around transcription start sites (TSS) (**Figures 3B, C**). PA+OA treatment significantly increased chromatin accessibility in 2,097 peaks genomic regions (**Figure 3D**). Pathway enrichment analysis of these differential peaks revealed significantly enriched pathways. (**Figures 3E, F**).

To identify key transcriptional regulators, we performed motif enrichment analysis of the PA+OA-induced accessible regions and integrated these data with RNA-seq results. This analysis revealed several candidate transcription factors, including *Cebpb*, *Elf1*, *Zbtb32*, and *Batf2*, that were significantly enriched and transcriptionally upregulated (**Figure 3G**). Among these, C/EBPβ was prioritized for further investigation due to reported association with lipid metabolism regulation.

Importantly, chromatin accessibility increased at the *Cd36* locus, with C/EBPβ binding detected upstream of the gene (**Figure 3H**). Immunofluorescence staining further confirmed increased nuclear C/EBPβ signals accompanied by enhanced lipid accumulation in PA+OA-treated cells (**Figure 3I**).

Together, these findings suggest that PA+OA induces epigenetic changes in macrophages, at least in part through C/EBPβ-mediated regulation, which facilitates *Cd36* upregulation and promotes foam cell formation.

### 3.4 Palmitic and oleic acids enhance CD36-mediated macrophage foam cell formation via C/EBPβ

**Figure 4:**
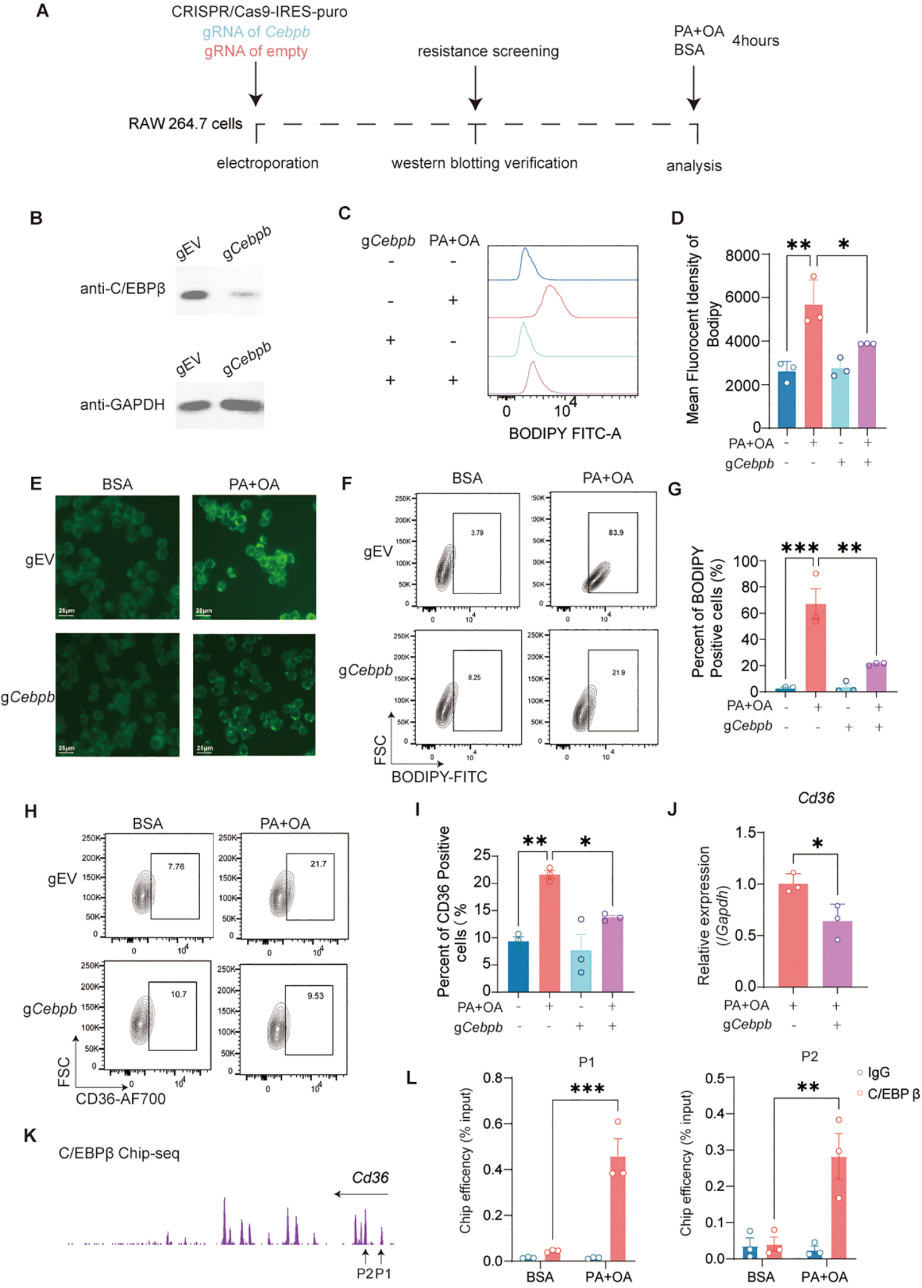
Palmitic and oleic acids enhance CD36-mediated macrophage foam cell formation via C/EBPβ. **(A)** Schematic representation of the experimental design for deficiency of C/EBPβ in RAW 264.7 cells. **(B)** Western blot validation of C/EBPβ deficiency in RAW 264.7 cells transfected with empty vector (gEV) or *Cebpb* gRNA (g*Cebpb*). **(C, D)** MFI of BODIPY in RAW 264.7 cells measured by flow cytometry. **(E)** Representative fluorescence images of BODIPY-stained RAW 264.7 cells transfected with gEV or g*Cebpb*, with or without PA+OA treatment. Scale bar, 25 μm. **(F, G)** Percentage of BODIPY-positive RAW 264.7 cells determined by flow cytometry. **(H, I)** Percentage of CD36-positive RAW 264.7 cells determined by flow cytometry. **(J)** Relative mRNA expression of *Cd36* in RAW 264.7 cells determined by qRT-PCR. **(K)** Representative ChIP-seq tracks showing C/EBPβ binding on the promoter region of *Cd36*. **(L)** Quantification of C/EBPβ binding efficiency at the promoter region of *Cd36* by ChIP-qPCR using specific primer sets (P1 and P2). Data are presented as mean ± SEM from independent experiments and analyzed using one-way ANOVA with Holm-Šídák’s multiple comparison test. **(J)** was performed using an unpaired t-test. ^*^p < 0.05, ^**^p < 0.01, ^***^p < 0.001. Three independent experiments were performed.

To validate the functional role of C/EBPβ in mediating FFA-induced CD36 expression and foam cell formation, we established *Cebpb*-deficient RAW 264.7 macrophages using CRISPR/Cas9 gene editing (**Figures 4A, B**). Compared with control cells transfected with empty vector, *Cebpb*-deficient macrophages showed a marked reduction in both the percentage of BODIPY-positive cells and MFI after PA+OA treatment (**Figures 4C–F**). Consistently, a substantial decrease in intracellular lipid accumulation in *Cebpb*-deficient macrophages (**Figure 4G**). Furthermore, *Cebpb* knockout significantly decreased the proportion of CD36-positive cells and suppressed *Cd36* mRNA expression in PA+OA-treated macrophages (**Figures 4H–J**).

To directly evaluate the transcriptional regulation of *Cd36* by C/EBPβ, ChIP assays were conducted. Based on ChIP-seq data, two primer sets targeting the transcription start region of *Cd36* were designed for validation (**Figure 4K**). ChIP-qPCR analysis revealed that PA+OA treatment significantly enhanced the binding of C/EBPβ to the *Cd36* promoter (**Figure 4L**), indicating that exposure to these fatty acids promotes C/EBPβ-mediated transcriptional activation of CD36.

Together, these findings demonstrate that C/EBPβ is a pivotal transcriptional regulator of CD36 and is required for PA+OA induced lipid uptake and foam cell formation in macrophages.

### 3.5 Loss of C/EBPβ alters the transcriptional landscape and inhibits foam cell formation

**Figure 5:**
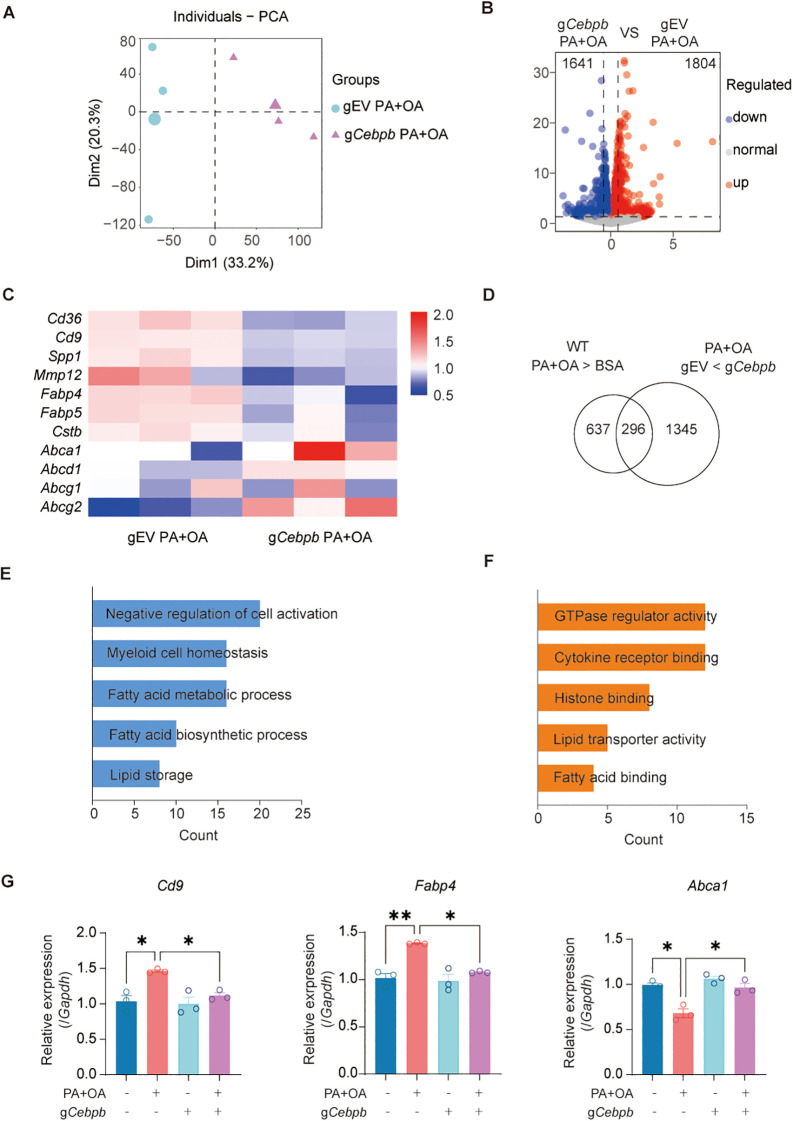
Loss of C/EBPβ alters the transcriptional landscape and inhibits foam cell formation **(A)** PCA of RNA-seq data from RAW 264.7 cells transfected with gEV or g*Cebpb* and treated with PA+OA. **(B)** Volcano plot showing differentially expressed genes between PA+OA-treated gEV and g*Cebpb* RAW 264.7 cells. **(C)** Heatmap of foam cell–associated genes derived from RNA-seq analysis. **(D)** Venn diagram showing the overlap between PA+OA-induced significantly upregulated genes and C/EBPβ loss–induced significantly downregulated genes. **(E, F)** GO enrichment analysis of overlapping genes, displaying enriched terms in the Biological Process **(E)** and Molecular Function **(F)** categories. **(G)** Relative mRNA expression of *Cd9*, *Fabp4*, *Abca1* determined by qRT-PCR. Data are presented as mean ± SEM. Statistical analysis was performed using one-way ANOVA with Tukey’s multiple comparisons test. ^*^p < 0.05, ^**^p < 0.01. Three independent experiments were performed.

To investigate the regulatory role of C/EBPβ in PA+OA-induced transcriptional responses, we performed RNA-sequencing on *Cebpb*-deficient and control RAW 264.7 cells following PA+OA treatment. PCA and hierarchical clustering revealed clear separation between the two groups, indicating a distinct transcriptional profile in the absence of *Cebpb* after PA+OA exposure (**Figure 5A**).

The volcano plot further highlighted these differences, showing that *Cebpb* deficiency resulted in the downregulation of 1,641 genes and the upregulation of 1,804 genes, reflecting a broad transcriptional reprogramming (**Figure 5B**). A heatmap focusing on key genes revealed that *Cd36*, which robustly upregulated upon PA+OA treatment in control cells, markedly reduced in *Cebpb*-deficient macrophages. In addition, foam cell-associated markers such as *Cd9*, *Spp1* significantly downregulated following *Cebpb* knockout, while *Mmp12* showed a decreasing trend that did not reach statistical significance. Interestingly, although fatty acid transporters *Fabp4* and *Fabp5* showed minimal change upon PA+OA stimulation, their expression was suppressed in the absence of *Cebpb*, suggesting impaired lipid uptake. Conversely, the expression of lipid efflux genes such as *Abca1* showed an increasing tendency following *Cebpb* knockout, indicating influenced macrophage lipid handling. (**Figure 5C**). A Venn diagram analysis revealed that *Cebpb* deficiency reversed a subset of genes upregulated by PA+OA treatment (**Figure 5D**). Notably, many of these genes are closely associated with lipid metabolism and transport (**Figures 5E, F**). Meanwhile, we detected the basal expression of *Cd9*, *Fabp4*, and *Abca1* in control and C/EBPβ-deficient macrophages with or without PA+OA stimulation by qRT-PCR (**Figure 5G**).

Collectively, these findings suggest that loss of *Cebpb* compromises the lipid uptake capacity of macrophages while promoting lipid efflux, thereby attenuating PA+OA induced foam cell formation.

Given that foam cells are key contributors to atherogenesis, we next analyzed public single-cell RNA-seq data from atherosclerotic lesions (**Supplementary Figure S3A**). We observed that *Cebpb* was broadly expressed in macrophage populations within plaques (**Supplementary Figures S3B, C**), suggesting its potential relevance across diverse macrophage states in the disease microenvironment.

### 3.6 C/EBPβ knockout inhibits FFA-induced lipid uptake in mouse BMDMs

**Figure 6:**
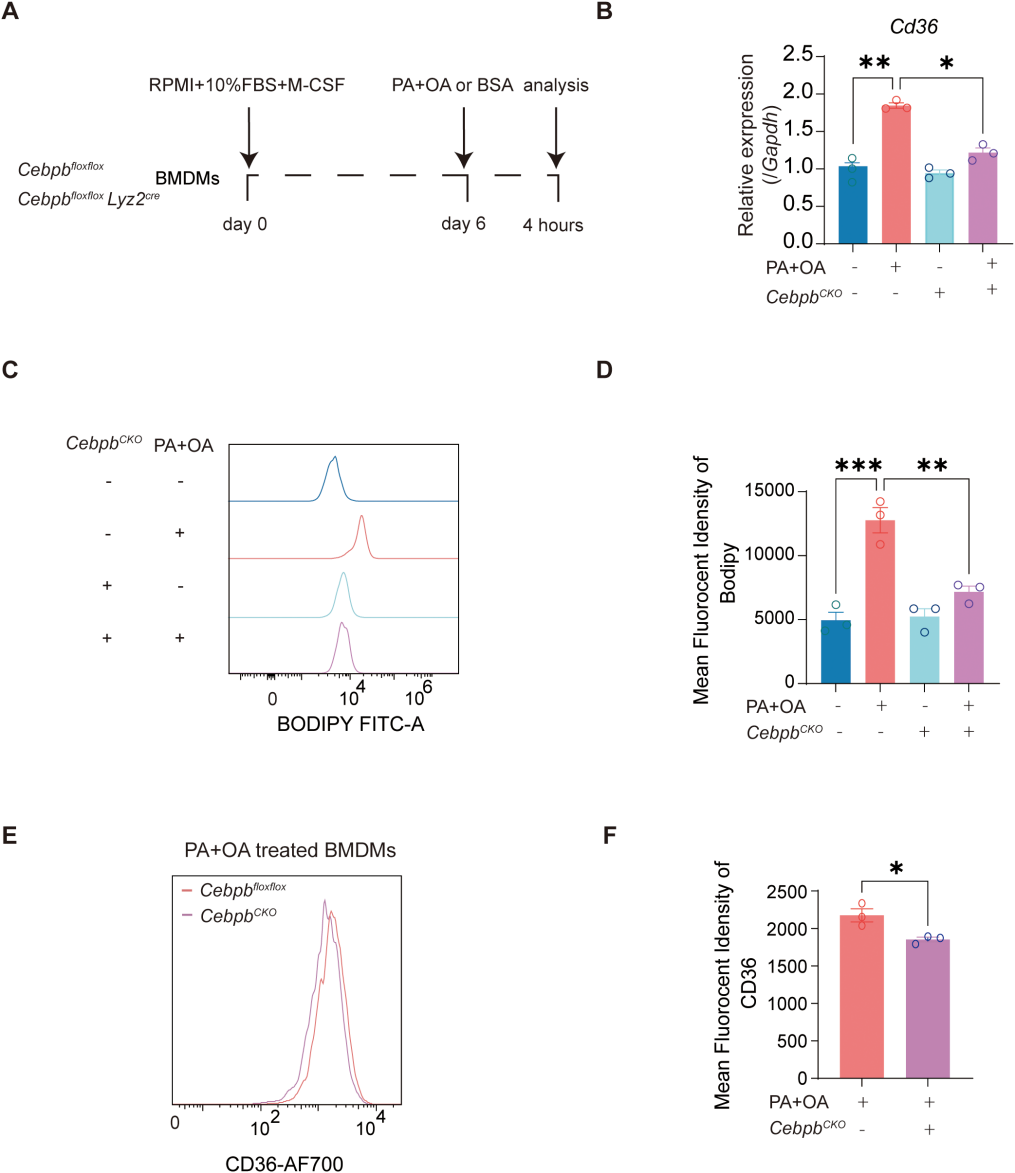
C/EBPβ deficiency suppresses palmitic and oleic acids induce lipid uptake and CD36 expression in mouse BMDMs. **(A)** Schematic representation of the experimental design using BMDMs from control and myeloid C/EBPβ-deficient mice. **(B)** Relative mRNA expression of *Cd36* in BMDMs determined by qRT-PCR. **(C, D)** MFI of BODIPY in BMDMs after treatment with PA and OA. **(E, F)** MFI of CD36 expression in BMDMs following PA and OA stimulation. Data are presented as mean ± SEM. Statistical analysis for **(B, D)** were performed using one-way ANOVA with Tukey’s multiple comparisons test. **(F)** was performed using an unpaired t-test. ^*^p < 0.05, ^**^p < 0.01, ^***^p < 0.001. Three independent experiments were performed.

Primary cells offer higher physiological fidelity and thus serve as an excellent ex vivo model for investigating cellular physiology, metabolism, signaling, and toxicity. To further validate our findings, we examined the role of C/EBPβ in mouse BMDMs (**Figure 6A**). Consistent with the observations in RAW 264.7-derived macrophages, treatment of myeloid C/EBPβ-deficient BMDMs with PA+OA markedly reduced *Cd36* mRNA expression (**Figure 6B**) and attenuated lipid uptake (**Figures 6C, D****).** Flow cytometry analysis further revealed that stimulation with PA+OA significantly suppressed CD36 expression in myeloid C/EBPβ-deficient BMDMs (**Figures 6E, F**). Collectively, these data from primary macrophages recapitulate the results obtained in RAW 264.7 cells and confirm the central role of C/EBPβ in regulating lipid accumulation via CD36-dependent signaling.

## 4 Discussion

In this study, we demonstrate that palmitic acid and oleic acid—the two most abundant dietary FFAs, promote macrophage foam cell formation primarily through C/EBPβ-dependent, CD36-mediated lipid uptake. Integrative multi-omics analysis revealed that FFAs induce extensive chromatin remodeling in macrophages, with C/EBPβ acting as a master transcriptional regulator directly activating *Cd36* expression. These findings offer novel insights into the molecular mechanisms driving excessive FFAs induced lipid accumulation in macrophages and suggest that targeting C/EBPβ may serve as a potential therapeutic strategy for metabolic diseases.

Our study provides critical mechanistic understanding of CD36 regulation—a multifunctional scavenger receptor orchestrating macrophage lipid uptake, transport, and homeostasis (39). While CD36 plays beneficial roles in physiological contexts such as fatty acid uptake for mitochondrial β-oxidation and antigen presentation, under conditions of chronic lipid overload, it contributes to pathological lipid retention, promoting foam cell formation and metabolic inflammation (4, 12, 30). CD36 deficiency has been associated with insulin resistance, whereas its overactivation aggravates lipid-related disorders including atherosclerosis (40–42). Our data suggest that epigenetic activation of *Cd36* via C/EBPβ-mediated chromatin remodeling is a key event in this maladaptive lipid response.

In this study, we used a combination of PA and OA to model fatty acid–induced foam cell formation in macrophages. PA and OA are among the most abundant circulating free fatty acids in humans and together represent a substantial proportion of plasma lipid pools (43, 44). Consistent with previous studies, combined PA and OA treatment promotes intracellular lipid accumulation while maintaining cellular viability, as OA facilitates triglyceride synthesis and lipid droplet formation and can partially buffer PA-induced lipotoxicity (45, 46). Nevertheless, macrophages *in vivo* are exposed to a far more complex lipid environment. Different lipid species vary in chain length, saturation, and biochemical modifications, which can influence lipid uptake, storage, and inflammatory signaling pathways (4, 32, 47). Future studies examining a broader range of lipid species will help further clarify how diverse lipid environments shape macrophage lipid metabolism and foam cell formation.

Importantly, our findings indicated C/EBPβ as a conserved regulator of lipid-induced macrophage activation. C/EBPβ has been implicated in lipid metabolism in various systems: it regulates neural metabolic function (48), adipocyte tissue homeostasis (28), facilitates surfactant clearance in alveolar macrophages (15), and as shown in previous work, is activated in adipose tissue macrophages following lipid-rich meals (27). The consistent co-activation of C/EBPβ and CD36 in lipid-rich environments suggests that this transcriptional axis represents an evolutionarily conserved program for sensing extracellular lipid overload—a common feature of many metabolic disorders characterized by tissue stress, cell death, and chronic inflammation. C/EBPβ may act as a lipid-responsive transcriptional sensor, or even a pattern recognition factor, that detects perturbations in tissue lipid homeostasis. Understanding the broader contexts in which this regulatory module operates may reveal novel, shared mechanisms underlying the pathogenesis of lipid-associated diseases (17, 49, 50).

Notably, C/EBPβ is known to exert pleiotropic functions in macrophage biology, contributing not only to inflammatory activation but also to tissue-repair programs (26, 51, 52). This dual role suggests that its function in atherosclerosis may be highly context- and stage-dependent. In early disease stages, where lipid overload and inflammatory signaling predominate, C/EBPβ-driven pathways may promote pathological lipid uptake and foam cell formation (53). In contrast, during later or resolving phases, C/EBPβ may participate in macrophage programs associated with tissue repair and recovery following injury (52). In this regard, our findings raise the possibility that selectively targeting the C/EBPβ–CD36 axis, rather than globally inhibiting C/EBPβ, may represent a more precise therapeutic strategy. Such an approach could potentially attenuate lipid accumulation and foam cell formation while preserving the beneficial roles of C/EBPβ in tissue repair. More broadly, future strategies aimed at temporally controlled, cell-type-specific, or pathway-selective modulation of C/EBPβ activity may be required to maximize therapeutic benefit while minimizing unintended effects.

Although tissue-resident macrophages exhibit unique gene expression profiles shaped by local cues, the conserved role for C/EBPβ in lipid handling across different disease contexts—including atherosclerosis, non-alcoholic fatty liver disease, and obesity-associated inflammation that warrants further investigated.

In summary, our findings identify C/EBPβ as a key mediator of FFA-induced foam cell formation through its direct regulation of CD36. This study establishes a mechanistic link between dietary lipid stress, epigenetic remodeling, and macrophage lipid accumulation. Future studies should evaluate whether pharmacological inhibition of C/EBPβ can attenuate lipid overload and inflammation *in vivo*. Additionally, elucidating how other metabolic cues integrate with C/EBPβ signaling will deepen our understanding of macrophage lipid homeostasis and offer new avenues for therapeutic intervention in metabolic diseases.

## 5 Conclusion

This study uncovers a novel regulatory mechanism by which FFAs promote CD36-mediated macrophage foam cell formation via C/EBPβ-driven epigenetic modifications. Deleting *Cebpb* disrupts this pathway, reducing lipid uptake and inflammatory monocyte activation *in vivo*. These findings highlight C/EBPβ as a potential target for therapeutic intervention in metabolic diseases characterized by dysregulated lipid metabolism.

## Data Availability

The dataset generated in this study is accessible at PRJNA1306332. Additional information required to reanalyze the data reported in this paper is available from the lead contact upon request.
