# Supplementary material for: Palmitic and oleic acids induce macrophage foam cell formation through C/EBPβ activation
Source: Front Immunol. 2026 Apr 29;17:1809059. doi: 10.3389/fimmu.2026.1809059 (PMC13167414; doi:10.3389/fimmu.2026.1809059)
Supplement: Supplementary file 1 [file DataSheet1.docx]

#### Supporting Information


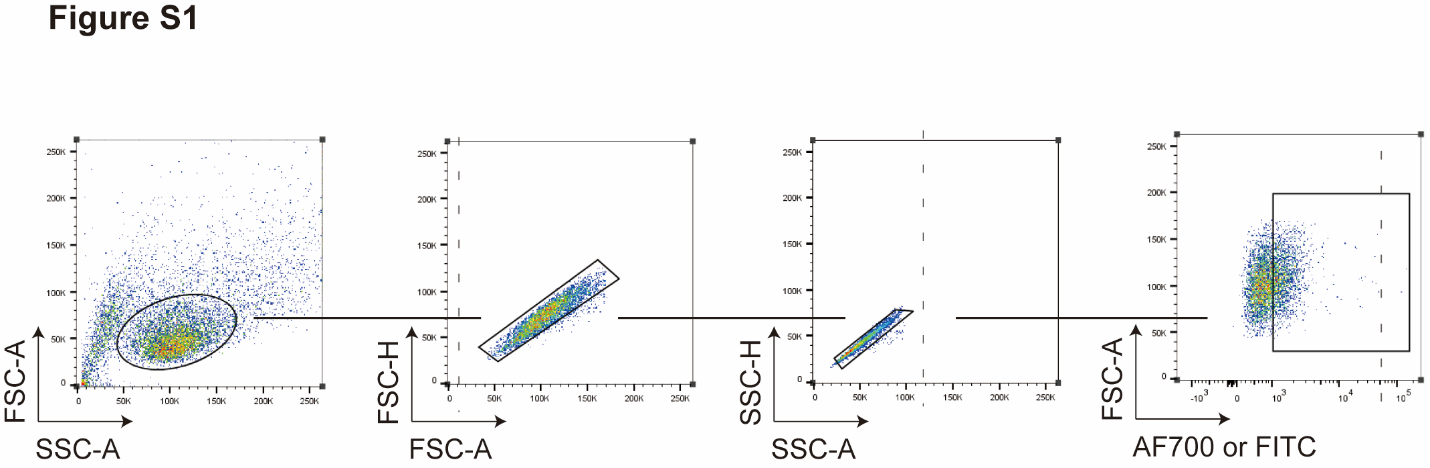
 **Figure S1. Representative gating strategies of flow cytometry analysis**


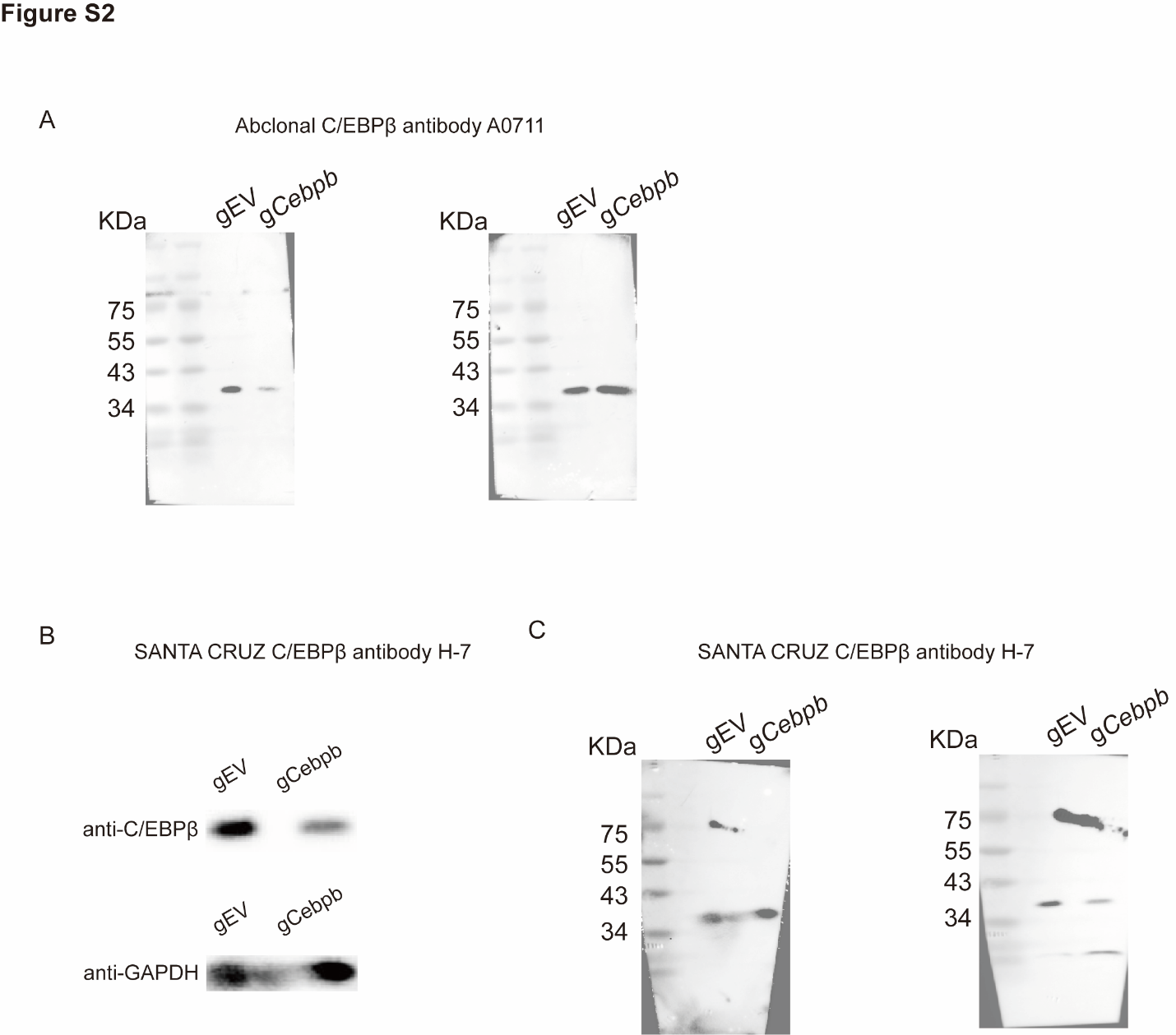


**Figure S2. Images of Western blot analysis.**

**(A)** Whole gel images of Figure 4B. **(B)** Western blot validation of C/EBPβ deficiency in RAW 264.7 cells transfected with gEV or g*Cebpb* by SANTA CRUZ C/EBPβ H-7. **(C)** Whole gel images of Figure S2B


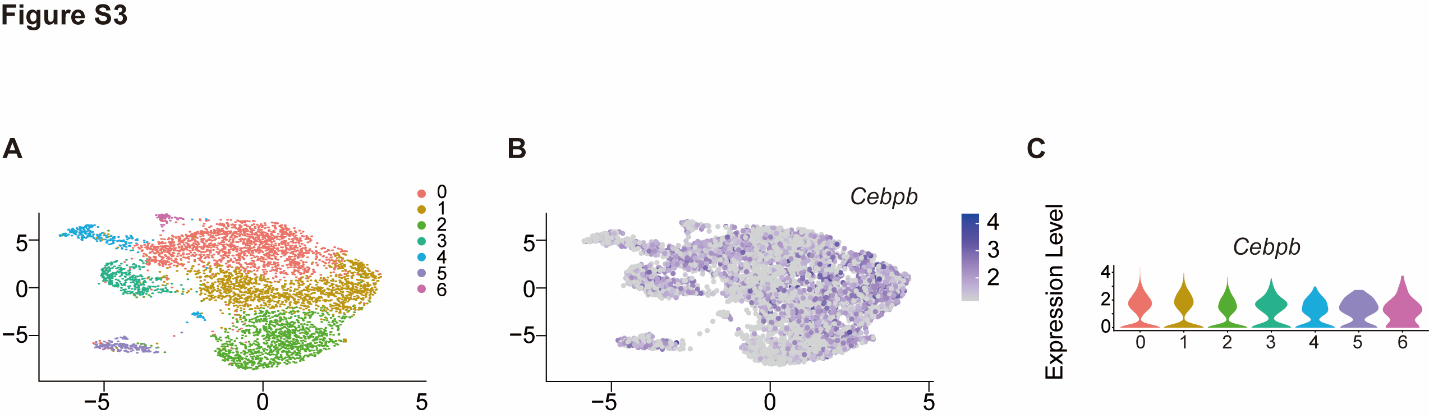


**Figure S3. C/EBPβ is broadly expressed in macrophage populations within atherosclerotic lesions**

**(A)** UMAP visualization of public single-cell RNA-seq data from atherosclerotic lesions, with cells colored by cluster identity. **(B)** Feature plots showing the expression of *Cebpb* across macrophage populations. **(C)** Violin plots illustrating the distribution of *Cebpb* expression across different macrophage clusters.

**Supplementary Tables**

**Table S1.** **Key resources table**

| **Reagent or resource** | | **Identifier** | **Source** | |
| --- | --- | --- | --- | --- |
| **Antibodies** | |  |  | |
| Alexa Fluor 700 CD36 Monoclonal Antibody (HM36) | | 56-0362-82 | Thermo Fisher | |
| C/EBPβ Rabbit pAb | | A0711 | Abclonal | |
| GAPDH Mouse mAb | | AC033 | Abclonal | |
| Goat anti-Rabbit IgG Secondary Antibody, HRP | | GB23303 | Servicebio | |
| Goat anti-Rabbit IgG Secondary Antibody, Alexa Fluor 488 | | A-11008 | Thermo Fisher | |
| C/EBPβ antibody (H-7) | | sc-7962 | SCBT | |
| Mouse IgG antibody | | sc-52336 | SCBT | |
| **Chemicals, kit and buffer** | |  |  | |
| Palmitic acid | | 232958 | MedChemExpress | |
| Oleic acid | | O1008 | SIGMA | |
| Bovine Serum Albumin V | | A8020 | Solarbio | |
| Sulfo-N-succinimidyl oleate | | HY-112847A | MedChemExpress | |
| Puromycin dihydrochloride | | HY-B1743A | MedChemExpress | |
| Dimethyl sulfoxide | | D8371 | Solarbio | |
| Dulbecco's Modified Eagle Medium | | PM150210 | Procell | |
| Fetal Bovine Serum | | 164210-50 | Procell | |
| Penicillin and streptomycin | | PB180120 | Procell | |
| Prestained Protein marker | | P1018 | Lablead | |
| WesternBright ECL HRP substrate | | K-12045-D50 | Advansta | |
| Fixable Viability Dye eFluor 780 | | 65-0865-14 | Thermo Fisher | |
| RPMI 1640 | | L210KJ | Basalmedia Technologies | |
| Fetal bovine serum for flow cytometry | | C4055L1050 | Life-ilab | |
| eBioscience Foxp3 / Transcription Factor Staining Buffer Set | | 00-5523-00 | Invitrogen | |
| IC fixation buffer | | 00-8222-49 | Invitrogen | |
| Lipid Droplets Green Fluorescence Assay Kit | | A076250512 | Beyotime | |
| Trizol | | 15596026 | Thermo Fisher | |
| Nebnext Ultra RNA Library Prep Kit for Illumina | | E7530 | New England Biolabs | |
| TruePrep DNA Library Prep kit V2 for Illumina | | TD501 | Vazyme | |
| Qiagen PCR purification kit | | 28104 | Qiagen | |
| NEBNext Ultra II Q5 Master Mix | | M0544 | New England Biolabs | |
| NovoNGS Index Kit for Illumina | | N239 | novoprotein | |
| Qiagen MiniElute Reaction Celanup Kit | | 28206 | Qiagen | |
| RNA-direct SYBR Green Real time PCR Master Mix | | QRT-201 100 | TOYOBO | |
| Silicon-on-Sapphire sensing plate (11-02, R Plane) | | FmSOS1010046S2FT05US | MTI Corporation | |
| Pierce 16% Formaldehyde (w/v), Methanol-free | | 28908 | Thermo Fisher | |
| Dynabeads Protein G for Immunoprecipitation | | 10003D | Thermo Fisher | |
| Cytokine Array C1 kit | | AAM-CYT-1-8 | Raybiotech | |
| Hieff qPCR SYBR Green Master Mix | | 11204ES08 | Yeansen | |
| **Software and Algorithms** | |  |  | |
| R (v4.2.1) | |  | <https://cran.rstudio.org/banner.shtml> | |
| HISAT (v2.2.1) | |  | <https://daehwankimlab.github.io/hisat2/download>/ | |
| Featurecounts (v2.0.1) | |  | <https://subread.sourceforge.net/> | |
| Limma v3.56.1 | |  | <https://kasperdanielhansen.github.io/genbioconductor/html/limma.html> | |
| Gseabase (v1.62.0) | |  | <https://github.com/Bioconductor/GSEABase> | |
| Clusterprofiler (v4.8.1) | |  | <https://github.com/YuLab-SMU/clusterProfiler> | |
| Trim-Galore (version 0.6.10) | |  | <https://github.com/FelixKrueger/TrimGalore> | |
| Bowtie2 (version 2.5.1) | |  | <https://github.com/BenLangmead/bowtie2> | |
| MACS2 (version 2.2.6) | |  | <https://pypi.org/project/MACS2/> | |
| Sambamba (version 1.01) | |  | <https://github.com/biod/sambamba> | |
| HOMER (version 4.10.4) | |  | <http://homer.ucsd.edu/homer/download.html> | |
| Integrative Genomics Viewer | |  | <https://igv.org/> | |
| DeepTools (version 3.4.1) | |  | <Https://github.com/deeptools/deepTools/> | |
| DESeq2 | |  | <https://github.com/thelovelab/DESeq2> | |
| Flow Jo (v.10.0.7) | |  | Becton Dickinson | |
| Prism 9 | |  | Graphpad | |
| Seurat R package (v2.6) | |  | <https://satijalab.org/seurat/> | |
| **Mice and Others** | |  |  | |
| C57BL6J mice | |  | Gempharmatech | |
| RAW 264.7 cell | | CL-0190 | Procell | |
| Lenticrisp v2 plasmid | | 52961 | Addgene | |
| **Oligos** |  | | |  |
| **Primers** | **Fw** | | | **Rv** |
| *Cd36* | GGACATTGAGATTCTTTTCCTCTG | | | GCAAAGGCATTGGCTGGAAGAAC |
| *Abca1* | GGAGCCTTTGTGGAACTCTTCC | | | CGCTCTCTTCAGCCACTTTGAG |
| *Fabp4* | TGAAATCACCGCAGACGACAGG | | | GCTTGTCACCATCTCGTTTTCTC |
| *Cd9* | CTGTGGCATAGCTGGTCCTTTG | | | AGACCTCACTGATGGCTTCAGG |
| *Cd36*_ChIP |  | | |  |
| P1 | ACTTCCCAGATTCAGATGGAGC | | | AGGCAATCGCTCTAACAGGC |
| P2 | ACCCCATGCTGCTCTGCTAT | | | ACAGAGCATTGGGAGTTCCTC |
| **gRNA** |  | | |  |
| *Cebpb* | GCTGCTTGAACAAGTTCCGC | | | |
